# Tris[6-(3,5-dimethyl-1*H*-pyrazol-1-yl)picolinato]gadolinium(III) methanol hemisolvate 2.5-hydrate

**DOI:** 10.1107/S1600536808001931

**Published:** 2008-01-25

**Authors:** Zhao Kai, Xian-Hong Yin, Feng Yu, Zhu Jie, Cui-Wu Lin

**Affiliations:** a College of Chemistry and Ecological Engineering, Guangxi University for Nationalities, Nanning 530006, People’s Republic of China; b College of Chemistry and Chemical Engineering, Guangxi University, Nanning 530004, People’s Republic of China

## Abstract

In the title complex, [Gd(C_11_H_10_N_3_O_2_)_3_]·0.5CH_4_O·2.5H_2_O, the Gd atom is coordinated by six N atoms and three O atoms derived from three tridentate monoanionic 6-(3,5-dimethyl-1*H*-pyrazol-1-yl)picolinate ligands. The mol­ecules are linked together *via* hydrogen bonds involving the solvent water and methanol mol­ecules.

## Related literature

For related literature, see: Zhao *et al.* (2007 ▶); Yin *et al.* (2007 ▶).
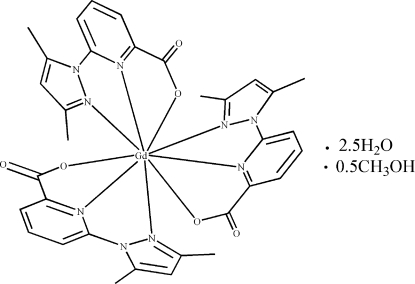


## Experimental

### 

#### Crystal data

[Gd(C_11_H_10_N_3_O_2_)_3_]·0.5CH_4_O·2.5H_2_O
*M* *_r_* = 866.97
Monoclinic, 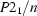

*a* = 15.5217 (18) Å
*b* = 16.5515 (19) Å
*c* = 16.434 (2) Å
β = 107.434 (2)°
*V* = 4028.0 (8) Å^3^
*Z* = 4
Mo *K*α radiation
μ = 1.71 mm^−1^
*T* = 298 (2) K
0.50 × 0.49 × 0.45 mm

#### Data collection

Bruker SMART 1000 diffractometer
Absorption correction: multi-scan (*SADABS*; Sheldrick, 1996 ▶) *T*_min_ = 0.483, *T*_max_ = 0.514 (expected range = 0.436–0.464)
19991 measured reflections
7101 independent reflections
5129 reflections with *I* > 2σ(*I*)
*R*_int_ = 0.037

#### Refinement

*R*[*F* ^2^ > 2σ(*F* ^2^)] = 0.044
*wR*(*F* ^2^) = 0.147
*S* = 1.08
7101 reflections
505 parameters
H-atom parameters constrained
Δρ_max_ = 1.34 e Å^−3^
Δρ_min_ = −1.00 e Å^−3^

### 

Data collection: *SMART* (Siemens, 1996 ▶); cell refinement: *SAINT* (Siemens, 1996 ▶); data reduction: *SAINT*; program(s) used to solve structure: *SHELXS97* (Sheldrick, 2008 ▶); program(s) used to refine structure: *SHELXL97* (Sheldrick, 2008 ▶); molecular graphics: *SHELXTL* (Sheldrick, 2008 ▶); software used to prepare material for publication: *SHELXTL*.

## Supplementary Material

Crystal structure: contains datablocks I, global. DOI: 10.1107/S1600536808001931/om2208sup1.cif

Structure factors: contains datablocks I. DOI: 10.1107/S1600536808001931/om2208Isup2.hkl

Additional supplementary materials:  crystallographic information; 3D view; checkCIF report

## Figures and Tables

**Table 1:** Hydrogen-bond geometry (Å, °)

*D*—H⋯*A*	*D*—H	H⋯*A*	*D*⋯*A*	*D*—H⋯*A*
O7—H7*D*⋯O8	0.85	2.23	3.074 (19)	172
O7—H7*E*⋯O2^i^	0.85	2.07	2.914 (13)	172
O8—H8*A*⋯O4^ii^	0.85	1.96	2.740 (11)	153
O8—H8*B*⋯O11	0.85	2.15	2.93 (2)	153
O9—H9*A*⋯O8	0.85	1.81	2.633 (18)	162
O9—H9*B*⋯O6^iii^	0.85	1.94	2.765 (17)	162
O10—H10*A*⋯O7	0.85	2.07	2.64 (2)	124
O11—H11⋯O2^i^	0.82	2.14	2.664 (17)	122
O12—H12*A*⋯O6^iv^	0.85	2.15	2.89 (3)	146
O12—H12*B*⋯O9^v^	0.84	1.74	2.49 (5)	147
O12—H12*B*⋯O8^v^	0.85	2.47	3.12 (4)	133

Symmetry codes: (i) 
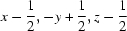
; (ii) 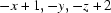; (iii) 
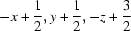
; (iv) 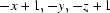; (v) 
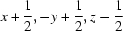
.

**Fig. 1.**
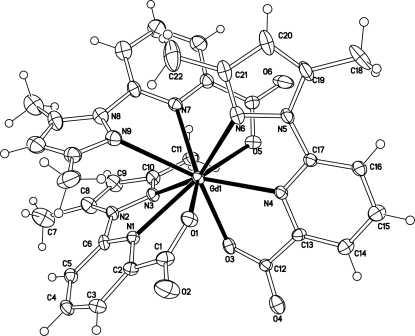
The structure of the title compound showing 50% probability displacement ellipsoids and the atom-numbering scheme. All of the solvate sites contain atoms that were refined at half occupancy.

**Fig. 2.**
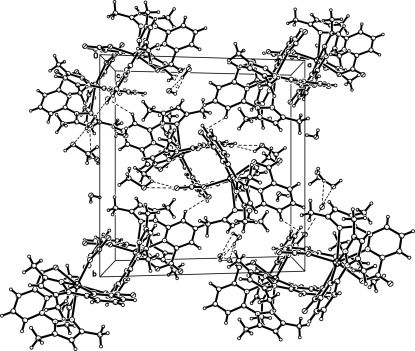
Crystal packing as viewed down the c-direction showing the hydrogen bonded interactions as dashed lines.

## supplementary crystallographic information

### Comment

Recently we reported the crystal structures of bis(6-(3,5-dimethyl-1*H*-pyrazol-1-yl)picolinato)zinc(II) trihydrate (Yin *et al.*, 2007) and bis[3-chloro-6-(3,5-dimethyl-1*H*-pyrazol-1-yl)picolinato]cobalt(II) 2.5- hydrate (Zhao *et al.*, 2007). As a continuation of these investigations, we report in this paper the crystal structure of tris(6-(3,5-dimethyl-1*H*-pyrazol-1-yl))picolinato)gadolinium(III) 2.5 hydrate 0.5 methanol solvate.

In the title complex, the gadolinium(III) ion is nine-coordinated by six N atoms and three O atoms derived from the tridentate ligands, 6-(3,5-dimethyl-1*H*-pyrazol-1-yl)picolinate (DPP); the angles around Gd(III) atom range from 60.34 (15) to 149.67 (16) °, the Gd—O bond lengths are 2.320 (4), 2.343 (4) and 2.372 (4) Å, The Gd—N distances range from 2.512 (5) to 2.742 (5) Å, *i.e.* normal values. The C1—C2 bond length is 1.520 (9) Å, being in the normal C—C ranges for gadolinium carboxylate complexes.

In the crystal structure, the oxygen atoms contribute to the formation of intermolecular hydrogen bonds involving the solvate molecules that link the complex into a three-dimensional network. (Fig. 2; for symmetry codes see Table 2).

### Experimental

6-(3,5-dimethyl-1*H*-pyrazol-1-yl)picolinic acid, and GdCl_3_. 6H_2_O were available commercially and were used without further purification. Equimolar 6-(3,5-dimethyl-1*H*-pyrazol-1-yl)picolinic acid (1 mmol, 217 mg) was dissolved in anhydrous ethyl methanol (AR, 99.9%) (15 ml). The mixture was stirred to give a clear solution, To this solution was added GdCl_3_.6H_2_O (0.33 mmol, 99 mg) in anhydrous methanol (10 ml). After keeping the resulting solution in air to evaporate about half of the solvents, colorless blocks of the title complex were formed. The crystals were isolated, washed with alcohol three times (yield, 75%). Elemental analysis: found: C, 46.31; H, 4.37; N, 14.46; calc. for C_67_H_74_Gd_2_N_18_O_18_: C, 46.41; H, 4.30; N, 14.54.

### Refinement

H atoms on C atoms were positoned geometrically and refined using a riding model with C—H = 0.96Å and *U*_iso_(H) = 1.2*U*_eq_(C). The water H atoms were located in difference Fourier maps and the O—H distances were constrained to 0.85 Å, with *U*_iso_(H) = 1.2*U*_eq_(O). The methanol O—H hydrogen atom was constrained with O—H distance of 0.82 Å. There are five waters of hydration sites and one methanol solvate site; all of these atoms were refined at half- occupancy. The largest difference map peaks are in the region of the disordered solvate molecules and probably represent alternative water and methanol sites that were not modeled.

### Crystal data

**Table:**

[Gd(C_11_H_10_N_3_O_2_)_3_]·0.5CH_4_O·2.5H_2_O	*F*_000_ = 1748
*M**_r_* = 866.97	*D*_x_ = 1.430 Mg m^−^^3^
Monoclinic, *P*2_1_/*n*	Mo *K*α radiation λ = 0.71073 Å
*a* = 15.5217 (18) Å	Cell parameters from 7251 reflections
*b* = 16.5515 (19) Å	θ = 1.6–25.0º
*c* = 16.434 (2) Å	µ = 1.71 mm^−^^1^
β = 107.434 (2)º	*T* = 298 (2) K
*V* = 4028.0 (8) Å^3^	Block, colorless
*Z* = 4	0.50 × 0.49 × 0.45 mm

### Data collection

**Table:**

Bruker SMART 1000 diffractometer	7101 independent reflections
Radiation source: fine-focus sealed tube	5129 reflections with *I* > 2σ(*I*)
Monochromator: graphite	*R*_int_ = 0.038
*T* = 298(2) K	θ_max_ = 25.0º
φ and ω scans	θ_min_ = 1.6º
Absorption correction: multi-scan(SADABS; Sheldrick, 1996)	*h* = −18→17
*T*_min_ = 0.483, *T*_max_ = 0.514	*k* = −19→19
19991 measured reflections	*l* = −15→19

### Refinement

**Table:**

Refinement on *F*^2^	Secondary atom site location: difference Fourier map
Least-squares matrix: full	Hydrogen site location: inferred from neighbouring sites
*R*[*F*^2^ > 2σ(*F*^2^)] = 0.044	H-atom parameters constrained
*wR*(*F*^2^) = 0.147	*w* = 1/[σ^2^(*F*_o_^2^) + (0.0899*P*)^2^ + 0.2214*P*] where *P* = (*F*_o_^2^ + 2*F*_c_^2^)/3
*S* = 1.08	(Δ/σ)_max_ = 0.001
7101 reflections	Δρ_max_ = 1.34 e Å^−^^3^
505 parameters	Δρ_min_ = −1.00 e Å^−^^3^
Primary atom site location: structure-invariant direct methods	Extinction correction: none

### Special details

**Table:**

Geometry. All e.s.d.'s (except the e.s.d. in the dihedral angle between two l.s. planes) are estimated using the full covariance matrix. The cell e.s.d.'s are taken into account individually in the estimation of e.s.d.'s in distances, angles and torsion angles; correlations between e.s.d.'s in cell parameters are only used when they are defined by crystal symmetry. An approximate (isotropic) treatment of cell e.s.d.'s is used for estimating e.s.d.'s involving l.s. planes.
Refinement. Refinement of *F*^2^ against ALL reflections. The weighted *R*-factor *wR* and goodness of fit *S* are based on *F*^2^, conventional *R*-factors *R* are based on *F*, with *F* set to zero for negative *F*^2^. The threshold expression of *F*^2^ > σ(*F*^2^) is used only for calculating *R*-factors(gt) *etc*. and is not relevant to the choice of reflections for refinement. *R*-factors based on *F*^2^ are statistically about twice as large as those based on *F*, and *R*- factors based on ALL data will be even larger.

### Fractional atomic coordinates and isotropic or equivalent isotropic displacement parameters (Å^2^)

**Table:**

	*x*	*y*	*z*	*U*_iso_*/*U*_eq_	Occ. (<1)
Gd1	0.704685 (17)	0.060270 (16)	0.866254 (18)	0.03314 (13)	
N1	0.8658 (3)	0.0884 (3)	0.9613 (3)	0.0352 (11)	
N2	0.9217 (3)	0.0719 (3)	0.8485 (3)	0.0372 (12)	
N3	0.8436 (3)	0.0310 (3)	0.8074 (3)	0.0410 (12)	
N4	0.5908 (3)	−0.0223 (3)	0.9109 (3)	0.0361 (11)	
N5	0.4775 (3)	0.0643 (3)	0.8416 (4)	0.0428 (13)	
N6	0.5442 (3)	0.1201 (3)	0.8435 (4)	0.0514 (14)	
N7	0.6714 (3)	0.1199 (3)	0.7172 (3)	0.0422 (12)	
N8	0.7776 (4)	0.2190 (3)	0.7700 (3)	0.0484 (13)	
N9	0.7582 (4)	0.2151 (3)	0.8470 (3)	0.0489 (13)	
O1	0.7175 (3)	0.1017 (3)	1.0057 (3)	0.0465 (11)	
O2	0.7941 (3)	0.1072 (5)	1.1439 (3)	0.0833 (19)	
O3	0.7600 (3)	−0.0609 (2)	0.9325 (3)	0.0439 (11)	
O4	0.7576 (3)	−0.1610 (3)	1.0214 (3)	0.0575 (12)	
O5	0.6111 (3)	−0.0179 (2)	0.7530 (3)	0.0459 (10)	
O6	0.5029 (4)	−0.0245 (3)	0.6291 (4)	0.0802 (18)	
O7	0.1858 (7)	0.2686 (7)	0.6923 (8)	0.090 (4)	0.50
H7D	0.2024	0.2721	0.7464	0.108*	0.50
H7E	0.2154	0.3030	0.6731	0.108*	0.50
O8	0.2573 (8)	0.2951 (7)	0.8861 (8)	0.100 (4)	0.50
H8A	0.2711	0.2522	0.9155	0.120*	0.50
H8B	0.3037	0.3124	0.8742	0.120*	0.50
O9	0.1011 (9)	0.3661 (10)	0.8144 (10)	0.125 (5)	0.50
H9A	0.1513	0.3492	0.8471	0.150*	0.50
H9B	0.0785	0.3991	0.8420	0.150*	0.50
O10	0.1065 (14)	0.1462 (12)	0.5951 (12)	0.191 (9)	0.50
H10A	0.1499	0.1791	0.6004	0.229*	0.50
H10B	0.0670	0.1558	0.5477	0.229*	0.50
O11	0.3711 (10)	0.3944 (12)	0.8122 (10)	0.131 (6)	0.50
H11	0.3671	0.4249	0.7719	0.197*	0.50
O12	0.692 (2)	0.033 (2)	0.417 (2)	0.35 (3)	0.50
H12A	0.6414	0.0138	0.4181	0.426*	0.50
H12B	0.6814	0.0732	0.3838	0.426*	0.50
C1	0.7885 (4)	0.1022 (4)	1.0680 (4)	0.0479 (16)	
C2	0.8756 (4)	0.0975 (4)	1.0439 (4)	0.0387 (14)	
C3	0.9603 (4)	0.1085 (4)	1.1044 (4)	0.0504 (17)	
H3	0.9661	0.1134	1.1622	0.060*	
C4	1.0346 (4)	0.1116 (4)	1.0750 (4)	0.0479 (16)	
H4	1.0916	0.1202	1.1133	0.057*	
C5	1.0253 (4)	0.1023 (4)	0.9908 (4)	0.0441 (15)	
H5	1.0753	0.1047	0.9708	0.053*	
C6	0.9389 (4)	0.0889 (3)	0.9345 (4)	0.0379 (14)	
C7	1.0654 (5)	0.1232 (6)	0.8168 (5)	0.086 (3)	
H7A	1.0587	0.1748	0.8408	0.129*	
H7B	1.0818	0.1308	0.7655	0.129*	
H7C	1.1118	0.0929	0.8571	0.129*	
C8	0.9764 (4)	0.0771 (5)	0.7964 (5)	0.059 (2)	
C9	0.9325 (5)	0.0386 (5)	0.7233 (5)	0.064 (2)	
H9	0.9529	0.0325	0.6760	0.077*	
C10	0.8515 (4)	0.0099 (4)	0.7313 (4)	0.0490 (16)	
C11	0.7833 (5)	−0.0415 (5)	0.6708 (5)	0.072 (2)	
H11A	0.7521	−0.0102	0.6216	0.108*	
H11B	0.7408	−0.0611	0.6982	0.108*	
H11C	0.8129	−0.0864	0.6535	0.108*	
C12	0.7212 (4)	−0.1057 (4)	0.9742 (4)	0.0368 (13)	
C13	0.6208 (4)	−0.0897 (4)	0.9568 (4)	0.0375 (13)	
C14	0.5651 (4)	−0.1426 (4)	0.9806 (4)	0.0456 (15)	
H14	0.5881	−0.1882	1.0128	0.055*	
C15	0.4736 (4)	−0.1264 (4)	0.9556 (5)	0.0560 (19)	
H15	0.4341	−0.1614	0.9707	0.067*	
C16	0.4410 (4)	−0.0585 (4)	0.9081 (5)	0.0493 (17)	
H16	0.3795	−0.0470	0.8903	0.059*	
C17	0.5028 (4)	−0.0078 (4)	0.8876 (4)	0.0389 (14)	
C18	0.3043 (5)	0.0523 (6)	0.7872 (7)	0.105 (4)	
H18A	0.2844	0.0573	0.8369	0.157*	
H18B	0.3122	−0.0038	0.7763	0.157*	
H18C	0.2600	0.0755	0.7390	0.157*	
C19	0.3945 (4)	0.0969 (5)	0.8020 (5)	0.063 (2)	
C20	0.4093 (5)	0.1734 (5)	0.7808 (5)	0.075 (2)	
H20	0.3653	0.2109	0.7539	0.090*	
C21	0.5023 (4)	0.1859 (4)	0.8064 (5)	0.0600 (19)	
C22	0.5512 (6)	0.2626 (5)	0.8014 (8)	0.108 (4)	
H22A	0.5166	0.3077	0.8110	0.162*	
H22B	0.5594	0.2673	0.7460	0.162*	
H22C	0.6091	0.2622	0.8441	0.162*	
C23	0.5688 (5)	0.0082 (4)	0.6795 (4)	0.0512 (17)	
C24	0.6078 (4)	0.0844 (4)	0.6530 (4)	0.0466 (16)	
C25	0.5839 (5)	0.1123 (5)	0.5715 (5)	0.066 (2)	
H25	0.5396	0.0860	0.5288	0.079*	
C26	0.6265 (5)	0.1809 (5)	0.5527 (5)	0.071 (2)	
H26	0.6115	0.2011	0.4974	0.085*	
C27	0.6910 (5)	0.2178 (5)	0.6176 (5)	0.063 (2)	
H27	0.7200	0.2641	0.6074	0.076*	
C28	0.7124 (4)	0.1845 (4)	0.6996 (4)	0.0480 (16)	
C29	0.8930 (5)	0.2784 (5)	0.7065 (6)	0.079 (3)	
H29A	0.9561	0.2902	0.7308	0.119*	
H29B	0.8638	0.3227	0.6712	0.119*	
H29C	0.8861	0.2303	0.6725	0.119*	
C30	0.8508 (5)	0.2659 (4)	0.7767 (5)	0.059 (2)	
C31	0.8787 (5)	0.2951 (4)	0.8578 (6)	0.066 (2)	
H31	0.9277	0.3291	0.8810	0.079*	
C32	0.8200 (5)	0.2644 (4)	0.8990 (5)	0.059 (2)	
C33	0.8156 (7)	0.2833 (5)	0.9858 (5)	0.086 (3)	
H33A	0.7685	0.2520	0.9974	0.128*	
H33B	0.8030	0.3398	0.9894	0.128*	
H33C	0.8723	0.2705	1.0270	0.128*	
C34	0.3849 (15)	0.4409 (15)	0.889 (2)	0.145 (13)	0.50
H34A	0.4129	0.4076	0.9375	0.218*	0.50
H34B	0.3277	0.4599	0.8926	0.218*	0.50
H34C	0.4231	0.4862	0.8879	0.218*	0.50

### Atomic displacement parameters (Å^2^)

**Table:**

	*U*^11^	*U*^22^	*U*^33^	*U*^12^	*U*^13^	*U*^23^
Gd1	0.02672 (18)	0.0401 (2)	0.0335 (2)	−0.00086 (11)	0.01038 (13)	0.00180 (13)
N1	0.030 (3)	0.035 (2)	0.042 (3)	−0.001 (2)	0.013 (2)	0.001 (2)
N2	0.027 (3)	0.048 (3)	0.040 (3)	0.001 (2)	0.015 (2)	0.004 (2)
N3	0.030 (3)	0.049 (3)	0.045 (3)	0.002 (2)	0.014 (2)	0.004 (3)
N4	0.029 (2)	0.047 (3)	0.033 (3)	0.001 (2)	0.010 (2)	0.000 (2)
N5	0.026 (2)	0.057 (3)	0.046 (3)	0.004 (2)	0.011 (2)	−0.003 (3)
N6	0.042 (3)	0.057 (3)	0.057 (4)	0.009 (3)	0.019 (3)	0.013 (3)
N7	0.041 (3)	0.047 (3)	0.037 (3)	0.001 (2)	0.010 (2)	0.007 (2)
N8	0.050 (3)	0.049 (3)	0.046 (3)	−0.002 (3)	0.015 (3)	0.012 (3)
N9	0.050 (3)	0.046 (3)	0.047 (3)	−0.002 (2)	0.009 (3)	0.006 (3)
O1	0.034 (2)	0.069 (3)	0.041 (3)	−0.004 (2)	0.016 (2)	−0.006 (2)
O2	0.062 (4)	0.145 (6)	0.049 (4)	−0.017 (3)	0.027 (3)	−0.021 (3)
O3	0.030 (2)	0.048 (2)	0.058 (3)	0.0023 (17)	0.020 (2)	0.011 (2)
O4	0.050 (3)	0.062 (3)	0.061 (3)	0.010 (2)	0.017 (2)	0.026 (3)
O5	0.049 (2)	0.047 (2)	0.041 (3)	−0.010 (2)	0.011 (2)	−0.002 (2)
O6	0.069 (4)	0.074 (3)	0.072 (4)	−0.017 (3)	−0.017 (3)	−0.013 (3)
O7	0.065 (7)	0.117 (10)	0.093 (9)	0.016 (6)	0.029 (6)	0.068 (8)
O8	0.091 (9)	0.095 (9)	0.130 (11)	0.010 (7)	0.058 (8)	0.068 (8)
O9	0.093 (10)	0.154 (14)	0.128 (13)	0.028 (10)	0.032 (9)	0.027 (11)
O10	0.21 (2)	0.19 (2)	0.138 (17)	−0.011 (16)	0.001 (15)	−0.008 (15)
O11	0.104 (11)	0.197 (17)	0.086 (11)	0.012 (11)	0.019 (9)	−0.038 (12)
O12	0.26 (4)	0.53 (6)	0.36 (4)	−0.24 (4)	0.23 (4)	−0.28 (5)
C1	0.049 (4)	0.058 (4)	0.039 (4)	−0.010 (3)	0.017 (3)	−0.007 (3)
C2	0.035 (3)	0.043 (3)	0.037 (4)	−0.006 (3)	0.010 (3)	−0.003 (3)
C3	0.047 (4)	0.056 (4)	0.043 (4)	−0.008 (3)	0.006 (3)	−0.003 (3)
C4	0.031 (3)	0.046 (4)	0.058 (5)	−0.002 (3)	0.001 (3)	−0.002 (3)
C5	0.033 (3)	0.043 (3)	0.057 (4)	−0.001 (3)	0.014 (3)	0.000 (3)
C6	0.030 (3)	0.032 (3)	0.054 (4)	0.006 (2)	0.016 (3)	0.014 (3)
C7	0.054 (5)	0.135 (8)	0.075 (6)	−0.033 (5)	0.027 (4)	0.015 (6)
C8	0.035 (4)	0.091 (5)	0.057 (5)	−0.003 (3)	0.025 (3)	0.011 (4)
C9	0.056 (5)	0.095 (6)	0.050 (5)	0.007 (4)	0.030 (4)	0.006 (4)
C10	0.047 (4)	0.067 (4)	0.040 (4)	0.006 (3)	0.023 (3)	−0.001 (3)
C11	0.061 (5)	0.091 (6)	0.061 (5)	−0.001 (4)	0.014 (4)	−0.027 (5)
C12	0.033 (3)	0.046 (3)	0.034 (3)	−0.003 (3)	0.013 (3)	−0.003 (3)
C13	0.035 (3)	0.048 (3)	0.033 (3)	−0.007 (3)	0.015 (3)	−0.006 (3)
C14	0.049 (4)	0.047 (4)	0.047 (4)	−0.006 (3)	0.023 (3)	−0.001 (3)
C15	0.049 (4)	0.066 (5)	0.066 (5)	−0.025 (3)	0.036 (4)	−0.014 (4)
C16	0.028 (3)	0.061 (4)	0.064 (5)	−0.005 (3)	0.020 (3)	−0.011 (4)
C17	0.030 (3)	0.050 (4)	0.036 (3)	0.002 (3)	0.009 (2)	−0.005 (3)
C18	0.030 (4)	0.144 (10)	0.119 (9)	0.004 (4)	−0.011 (5)	0.029 (7)
C19	0.026 (3)	0.091 (6)	0.066 (5)	0.017 (4)	0.006 (3)	0.004 (5)
C20	0.047 (4)	0.086 (6)	0.089 (6)	0.028 (4)	0.018 (4)	0.027 (5)
C21	0.046 (4)	0.067 (5)	0.068 (5)	0.022 (4)	0.020 (3)	0.017 (4)
C22	0.090 (7)	0.067 (6)	0.182 (12)	0.028 (5)	0.064 (7)	0.059 (7)
C23	0.054 (4)	0.051 (4)	0.045 (4)	0.004 (3)	0.009 (3)	−0.011 (3)
C24	0.043 (4)	0.055 (4)	0.040 (4)	0.009 (3)	0.009 (3)	0.004 (3)
C25	0.076 (5)	0.072 (5)	0.041 (4)	0.005 (4)	0.003 (4)	0.007 (4)
C26	0.080 (5)	0.083 (6)	0.042 (4)	0.003 (5)	0.008 (4)	0.015 (4)
C27	0.073 (5)	0.066 (5)	0.058 (5)	0.009 (4)	0.031 (4)	0.026 (4)
C28	0.042 (3)	0.055 (4)	0.048 (4)	0.007 (3)	0.014 (3)	0.013 (3)
C29	0.064 (5)	0.082 (6)	0.095 (7)	−0.012 (4)	0.029 (5)	0.031 (5)
C30	0.050 (4)	0.052 (4)	0.080 (6)	0.003 (3)	0.025 (4)	0.025 (4)
C31	0.064 (5)	0.051 (4)	0.074 (6)	−0.015 (4)	0.007 (4)	0.013 (4)
C32	0.068 (5)	0.045 (4)	0.052 (4)	0.000 (3)	−0.003 (4)	0.009 (3)
C33	0.131 (8)	0.057 (5)	0.053 (5)	−0.012 (5)	0.004 (5)	−0.008 (4)
C34	0.055 (13)	0.15 (3)	0.21 (4)	−0.018 (13)	0.014 (17)	0.05 (2)

### Geometric parameters (Å, °)

**Table:**

Gd1—O3	2.320 (4)	C7—C8	1.525 (9)
Gd1—O1	2.343 (4)	C7—H7A	0.9600
Gd1—O5	2.372 (4)	C7—H7B	0.9600
Gd1—N4	2.512 (5)	C7—H7C	0.9600
Gd1—N7	2.546 (5)	C8—C9	1.349 (10)
Gd1—N1	2.563 (4)	C9—C10	1.386 (9)
Gd1—N6	2.601 (5)	C9—H9	0.9300
Gd1—N3	2.659 (5)	C10—C11	1.483 (9)
Gd1—N9	2.742 (5)	C11—H11A	0.9600
N1—C2	1.329 (7)	C11—H11B	0.9600
N1—C6	1.334 (7)	C11—H11C	0.9600
N2—N3	1.377 (7)	C12—C13	1.522 (8)
N2—C8	1.377 (8)	C13—C14	1.367 (8)
N2—C6	1.387 (8)	C14—C15	1.380 (9)
N3—C10	1.339 (8)	C14—H14	0.9300
N4—C17	1.326 (7)	C15—C16	1.375 (9)
N4—C13	1.348 (8)	C15—H15	0.9300
N5—C19	1.368 (8)	C16—C17	1.391 (9)
N5—N6	1.381 (7)	C16—H16	0.9300
N5—C17	1.403 (7)	C18—C19	1.536 (11)
N6—C21	1.321 (8)	C18—H18A	0.9600
N7—C28	1.321 (8)	C18—H18B	0.9600
N7—C24	1.345 (8)	C18—H18C	0.9600
N8—C30	1.353 (9)	C19—C20	1.351 (11)
N8—N9	1.388 (8)	C20—C21	1.393 (10)
N8—C28	1.409 (8)	C20—H20	0.9300
N9—C32	1.350 (8)	C21—C22	1.494 (11)
O1—C1	1.260 (7)	C22—H22A	0.9600
O2—C1	1.226 (8)	C22—H22B	0.9600
O3—C12	1.275 (7)	C22—H22C	0.9600
O4—C12	1.223 (7)	C23—C24	1.518 (10)
O5—C23	1.265 (7)	C24—C25	1.359 (9)
O6—C23	1.232 (7)	C25—C26	1.395 (11)
O7—H7D	0.8500	C25—H25	0.9300
O7—H7E	0.8501	C26—C27	1.368 (10)
O8—H8A	0.8500	C26—H26	0.9300
O8—H8B	0.8501	C27—C28	1.400 (9)
O9—H9A	0.8500	C27—H27	0.9300
O9—H9B	0.8500	C29—C30	1.502 (11)
O10—H10A	0.8500	C29—H29A	0.9600
O10—H10B	0.8499	C29—H29B	0.9600
O11—C34	1.44 (3)	C29—H29C	0.9600
O11—H11	0.8200	C30—C31	1.361 (11)
O12—H12A	0.8501	C31—C32	1.385 (11)
O12—H12B	0.8400	C31—H31	0.9300
C1—C2	1.520 (9)	C32—C33	1.482 (11)
C2—C3	1.403 (8)	C33—H33A	0.9600
C3—C4	1.379 (9)	C33—H33B	0.9600
C3—H3	0.9300	C33—H33C	0.9600
C4—C5	1.357 (9)	C34—H34A	0.9600
C4—H4	0.9300	C34—H34B	0.9600
C5—C6	1.400 (8)	C34—H34C	0.9600
C5—H5	0.9300		
			
O3—Gd1—O1	83.26 (16)	N2—C8—C7	125.5 (7)
O3—Gd1—O5	87.15 (14)	C8—C9—C10	108.0 (6)
O1—Gd1—O5	141.23 (14)	C8—C9—H9	126.0
O3—Gd1—N4	65.55 (14)	C10—C9—H9	126.0
O1—Gd1—N4	74.60 (15)	N3—C10—C9	109.7 (6)
O5—Gd1—N4	67.26 (15)	N3—C10—C11	121.9 (6)
O3—Gd1—N7	136.58 (16)	C9—C10—C11	128.2 (7)
O1—Gd1—N7	139.55 (17)	C10—C11—H11A	109.5
O5—Gd1—N7	63.68 (15)	C10—C11—H11B	109.5
N4—Gd1—N7	122.31 (15)	H11A—C11—H11B	109.5
O3—Gd1—N1	73.43 (15)	C10—C11—H11C	109.5
O1—Gd1—N1	64.52 (14)	H11A—C11—H11C	109.5
O5—Gd1—N1	146.42 (15)	H11B—C11—H11C	109.5
N4—Gd1—N1	124.37 (15)	O4—C12—O3	125.0 (5)
N7—Gd1—N1	113.28 (16)	O4—C12—C13	120.0 (5)
O3—Gd1—N6	126.86 (16)	O3—C12—C13	114.8 (5)
O1—Gd1—N6	79.98 (16)	N4—C13—C14	123.2 (5)
O5—Gd1—N6	76.13 (17)	N4—C13—C12	114.2 (5)
N4—Gd1—N6	61.43 (16)	C14—C13—C12	122.4 (6)
N7—Gd1—N6	78.75 (17)	C13—C14—C15	118.2 (6)
N1—Gd1—N6	137.39 (17)	C13—C14—H14	120.9
O3—Gd1—N3	77.70 (15)	C15—C14—H14	120.9
O1—Gd1—N3	124.68 (15)	C16—C15—C14	119.9 (6)
O5—Gd1—N3	89.28 (15)	C16—C15—H15	120.0
N4—Gd1—N3	136.50 (15)	C14—C15—H15	120.0
N7—Gd1—N3	70.93 (16)	C15—C16—C17	117.9 (6)
N1—Gd1—N3	60.34 (15)	C15—C16—H16	121.1
N6—Gd1—N3	149.67 (16)	C17—C16—H16	121.1
O3—Gd1—N9	141.25 (15)	N4—C17—C16	123.1 (6)
O1—Gd1—N9	84.22 (16)	N4—C17—N5	114.1 (5)
O5—Gd1—N9	123.70 (15)	C16—C17—N5	122.8 (5)
N4—Gd1—N9	143.76 (16)	C19—C18—H18A	109.5
N7—Gd1—N9	60.52 (16)	C19—C18—H18B	109.5
N1—Gd1—N9	68.06 (15)	H18A—C18—H18B	109.5
N6—Gd1—N9	86.43 (16)	C19—C18—H18C	109.5
N3—Gd1—N9	79.74 (16)	H18A—C18—H18C	109.5
C2—N1—C6	119.2 (5)	H18B—C18—H18C	109.5
C2—N1—Gd1	115.7 (4)	C20—C19—N5	106.5 (6)
C6—N1—Gd1	124.8 (4)	C20—C19—C18	128.8 (7)
N3—N2—C8	110.0 (5)	N5—C19—C18	124.7 (7)
N3—N2—C6	118.2 (5)	C19—C20—C21	107.6 (6)
C8—N2—C6	130.9 (5)	C19—C20—H20	126.2
C10—N3—N2	105.9 (5)	C21—C20—H20	126.2
C10—N3—Gd1	134.3 (4)	N6—C21—C20	109.8 (7)
N2—N3—Gd1	115.0 (4)	N6—C21—C22	122.6 (6)
C17—N4—C13	117.7 (5)	C20—C21—C22	127.4 (7)
C17—N4—Gd1	124.8 (4)	C21—C22—H22A	109.5
C13—N4—Gd1	117.3 (3)	C21—C22—H22B	109.5
C19—N5—N6	109.9 (6)	H22A—C22—H22B	109.5
C19—N5—C17	131.5 (6)	C21—C22—H22C	109.5
N6—N5—C17	118.0 (5)	H22A—C22—H22C	109.5
C21—N6—N5	106.2 (5)	H22B—C22—H22C	109.5
C21—N6—Gd1	134.3 (5)	O6—C23—O5	125.5 (7)
N5—N6—Gd1	115.4 (3)	O6—C23—C24	119.6 (6)
C28—N7—C24	118.1 (5)	O5—C23—C24	114.9 (5)
C28—N7—Gd1	123.5 (4)	N7—C24—C25	123.0 (7)
C24—N7—Gd1	118.4 (4)	N7—C24—C23	113.5 (5)
C30—N8—N9	111.0 (6)	C25—C24—C23	123.4 (6)
C30—N8—C28	132.7 (6)	C24—C25—C26	119.2 (7)
N9—N8—C28	115.6 (5)	C24—C25—H25	120.4
C32—N9—N8	104.0 (6)	C26—C25—H25	120.4
C32—N9—Gd1	132.3 (4)	C27—C26—C25	118.4 (7)
N8—N9—Gd1	107.7 (4)	C27—C26—H26	120.8
C1—O1—Gd1	126.5 (4)	C25—C26—H26	120.8
C12—O3—Gd1	125.9 (4)	C26—C27—C28	118.8 (7)
C23—O5—Gd1	125.7 (4)	C26—C27—H27	120.6
H7D—O7—H7E	108.2	C28—C27—H27	120.6
H8A—O8—H8B	109.0	N7—C28—C27	122.6 (6)
H9A—O9—H9B	108.8	N7—C28—N8	115.0 (5)
H10A—O10—H10B	107.8	C27—C28—N8	122.4 (6)
C34—O11—H11	109.5	C30—C29—H29A	109.5
H12A—O12—H12B	107.6	C30—C29—H29B	109.5
O2—C1—O1	127.3 (7)	H29A—C29—H29B	109.5
O2—C1—C2	118.1 (6)	C30—C29—H29C	109.5
O1—C1—C2	114.6 (6)	H29A—C29—H29C	109.5
N1—C2—C3	122.3 (6)	H29B—C29—H29C	109.5
N1—C2—C1	115.8 (5)	N8—C30—C31	107.3 (7)
C3—C2—C1	121.8 (6)	N8—C30—C29	124.4 (8)
C4—C3—C2	117.5 (6)	C31—C30—C29	128.3 (7)
C4—C3—H3	121.2	C30—C31—C32	106.6 (7)
C2—C3—H3	121.2	C30—C31—H31	126.7
C5—C4—C3	120.5 (6)	C32—C31—H31	126.7
C5—C4—H4	119.7	N9—C32—C31	111.0 (7)
C3—C4—H4	119.7	N9—C32—C33	120.5 (8)
C4—C5—C6	118.7 (6)	C31—C32—C33	128.3 (7)
C4—C5—H5	120.6	C32—C33—H33A	109.5
C6—C5—H5	120.6	C32—C33—H33B	109.5
N1—C6—N2	114.3 (5)	H33A—C33—H33B	109.5
N1—C6—C5	121.6 (6)	C32—C33—H33C	109.5
N2—C6—C5	124.0 (6)	H33A—C33—H33C	109.5
C8—C7—H7A	109.5	H33B—C33—H33C	109.5
C8—C7—H7B	109.5	O11—C34—H34A	109.5
H7A—C7—H7B	109.5	O11—C34—H34B	109.5
C8—C7—H7C	109.5	H34A—C34—H34B	109.5
H7A—C7—H7C	109.5	O11—C34—H34C	109.5
H7B—C7—H7C	109.5	H34A—C34—H34C	109.5
C9—C8—N2	106.4 (6)	H34B—C34—H34C	109.5
C9—C8—C7	127.9 (7)		
			
O3—Gd1—N1—C2	−78.6 (4)	N1—Gd1—O3—C12	128.0 (5)
O1—Gd1—N1—C2	11.7 (4)	N6—Gd1—O3—C12	−9.5 (5)
O5—Gd1—N1—C2	−135.8 (4)	N3—Gd1—O3—C12	−169.7 (5)
N4—Gd1—N1—C2	−35.2 (5)	N9—Gd1—O3—C12	134.6 (4)
N7—Gd1—N1—C2	147.2 (4)	O3—Gd1—O5—C23	−165.5 (5)
N6—Gd1—N1—C2	48.3 (5)	O1—Gd1—O5—C23	119.0 (5)
N3—Gd1—N1—C2	−163.7 (5)	N4—Gd1—O5—C23	129.9 (5)
N9—Gd1—N1—C2	105.9 (4)	N7—Gd1—O5—C23	−18.7 (5)
O3—Gd1—N1—C6	96.7 (5)	N1—Gd1—O5—C23	−111.7 (5)
O1—Gd1—N1—C6	−173.0 (5)	N6—Gd1—O5—C23	65.4 (5)
O5—Gd1—N1—C6	39.5 (6)	N3—Gd1—O5—C23	−87.7 (5)
N4—Gd1—N1—C6	140.1 (4)	N9—Gd1—O5—C23	−10.6 (6)
N7—Gd1—N1—C6	−37.5 (5)	Gd1—O1—C1—O2	−163.4 (7)
N6—Gd1—N1—C6	−136.4 (4)	Gd1—O1—C1—C2	17.6 (8)
N3—Gd1—N1—C6	11.6 (4)	C6—N1—C2—C3	0.6 (9)
N9—Gd1—N1—C6	−78.9 (5)	Gd1—N1—C2—C3	176.1 (5)
C8—N2—N3—C10	1.2 (7)	C6—N1—C2—C1	175.8 (5)
C6—N2—N3—C10	−169.1 (5)	Gd1—N1—C2—C1	−8.6 (7)
C8—N2—N3—Gd1	−157.8 (4)	O2—C1—C2—N1	176.9 (7)
C6—N2—N3—Gd1	31.9 (6)	O1—C1—C2—N1	−4.1 (9)
O3—Gd1—N3—C10	109.9 (6)	O2—C1—C2—C3	−7.8 (10)
O1—Gd1—N3—C10	−177.4 (6)	O1—C1—C2—C3	171.2 (6)
O5—Gd1—N3—C10	22.6 (6)	N1—C2—C3—C4	1.7 (9)
N4—Gd1—N3—C10	77.5 (6)	C1—C2—C3—C4	−173.3 (6)
N7—Gd1—N3—C10	−39.7 (6)	C2—C3—C4—C5	−1.7 (9)
N1—Gd1—N3—C10	−172.4 (7)	C3—C4—C5—C6	−0.4 (9)
N6—Gd1—N3—C10	−37.6 (8)	C2—N1—C6—N2	174.7 (5)
N9—Gd1—N3—C10	−101.9 (6)	Gd1—N1—C6—N2	−0.4 (7)
O3—Gd1—N3—N2	−98.9 (4)	C2—N1—C6—C5	−2.8 (9)
O1—Gd1—N3—N2	−26.2 (4)	Gd1—N1—C6—C5	−178.0 (4)
O5—Gd1—N3—N2	173.8 (4)	N3—N2—C6—N1	−21.7 (7)
N4—Gd1—N3—N2	−131.3 (4)	C8—N2—C6—N1	170.5 (6)
N7—Gd1—N3—N2	111.5 (4)	N3—N2—C6—C5	155.8 (5)
N1—Gd1—N3—N2	−21.2 (3)	C8—N2—C6—C5	−12.0 (10)
N6—Gd1—N3—N2	113.6 (4)	C4—C5—C6—N1	2.8 (9)
N9—Gd1—N3—N2	49.3 (4)	C4—C5—C6—N2	−174.5 (6)
O3—Gd1—N4—C17	−168.9 (5)	N3—N2—C8—C9	−0.5 (8)
O1—Gd1—N4—C17	101.6 (5)	C6—N2—C8—C9	168.1 (6)
O5—Gd1—N4—C17	−71.3 (4)	N3—N2—C8—C7	175.3 (7)
N7—Gd1—N4—C17	−37.8 (5)	C6—N2—C8—C7	−16.1 (12)
N1—Gd1—N4—C17	144.8 (4)	N2—C8—C9—C10	−0.3 (9)
N6—Gd1—N4—C17	14.8 (4)	C7—C8—C9—C10	−176.0 (8)
N3—Gd1—N4—C17	−133.9 (4)	N2—N3—C10—C9	−1.3 (7)
N9—Gd1—N4—C17	45.1 (6)	Gd1—N3—C10—C9	151.6 (5)
O3—Gd1—N4—C13	6.0 (4)	N2—N3—C10—C11	174.9 (6)
O1—Gd1—N4—C13	−83.5 (4)	Gd1—N3—C10—C11	−32.1 (10)
O5—Gd1—N4—C13	103.6 (4)	C8—C9—C10—N3	1.0 (9)
N7—Gd1—N4—C13	137.1 (4)	C8—C9—C10—C11	−174.9 (7)
N1—Gd1—N4—C13	−40.3 (5)	Gd1—O3—C12—O4	−166.0 (5)
N6—Gd1—N4—C13	−170.3 (5)	Gd1—O3—C12—C13	18.1 (7)
N3—Gd1—N4—C13	41.1 (5)	C17—N4—C13—C14	−0.5 (9)
N9—Gd1—N4—C13	−139.9 (4)	Gd1—N4—C13—C14	−175.8 (5)
C19—N5—N6—C21	0.6 (8)	C17—N4—C13—C12	174.9 (5)
C17—N5—N6—C21	−171.4 (6)	Gd1—N4—C13—C12	−0.4 (6)
C19—N5—N6—Gd1	−159.8 (5)	O4—C12—C13—N4	173.6 (6)
C17—N5—N6—Gd1	28.2 (7)	O3—C12—C13—N4	−10.2 (7)
O3—Gd1—N6—C21	−178.4 (6)	O4—C12—C13—C14	−10.9 (9)
O1—Gd1—N6—C21	108.0 (7)	O3—C12—C13—C14	165.2 (6)
O5—Gd1—N6—C21	−102.8 (7)	N4—C13—C14—C15	0.7 (9)
N4—Gd1—N6—C21	−174.2 (7)	C12—C13—C14—C15	−174.3 (6)
N7—Gd1—N6—C21	−37.4 (7)	C13—C14—C15—C16	−0.2 (10)
N1—Gd1—N6—C21	74.9 (7)	C14—C15—C16—C17	−0.5 (10)
N3—Gd1—N6—C21	−39.4 (8)	C13—N4—C17—C16	−0.2 (9)
N9—Gd1—N6—C21	23.2 (7)	Gd1—N4—C17—C16	174.7 (5)
O3—Gd1—N6—N5	−25.1 (5)	C13—N4—C17—N5	178.7 (5)
O1—Gd1—N6—N5	−98.7 (4)	Gd1—N4—C17—N5	−6.4 (7)
O5—Gd1—N6—N5	50.5 (4)	C15—C16—C17—N4	0.7 (10)
N4—Gd1—N6—N5	−20.9 (4)	C15—C16—C17—N5	−178.1 (6)
N7—Gd1—N6—N5	115.9 (4)	C19—N5—C17—N4	174.7 (7)
N1—Gd1—N6—N5	−131.9 (4)	N6—N5—C17—N4	−15.4 (8)
N3—Gd1—N6—N5	113.9 (5)	C19—N5—C17—C16	−6.4 (11)
N9—Gd1—N6—N5	176.5 (4)	N6—N5—C17—C16	163.5 (6)
O3—Gd1—N7—C28	−117.8 (5)	N6—N5—C19—C20	−1.2 (9)
O1—Gd1—N7—C28	49.9 (6)	C17—N5—C19—C20	169.4 (7)
O5—Gd1—N7—C28	−170.6 (5)	N6—N5—C19—C18	179.2 (8)
N4—Gd1—N7—C28	154.8 (5)	C17—N5—C19—C18	−10.2 (13)
N1—Gd1—N7—C28	−27.6 (5)	N5—C19—C20—C21	1.3 (10)
N6—Gd1—N7—C28	109.4 (5)	C18—C19—C20—C21	−179.2 (9)
N3—Gd1—N7—C28	−71.7 (5)	N5—N6—C21—C20	0.2 (8)
N9—Gd1—N7—C28	17.1 (5)	Gd1—N6—C21—C20	155.2 (6)
O3—Gd1—N7—C24	63.2 (5)	N5—N6—C21—C22	175.4 (8)
O1—Gd1—N7—C24	−129.2 (4)	Gd1—N6—C21—C22	−29.6 (12)
O5—Gd1—N7—C24	10.3 (4)	C19—C20—C21—N6	−0.9 (10)
N4—Gd1—N7—C24	−24.3 (5)	C19—C20—C21—C22	−175.9 (9)
N1—Gd1—N7—C24	153.4 (4)	Gd1—O5—C23—O6	−159.2 (6)
N6—Gd1—N7—C24	−69.6 (5)	Gd1—O5—C23—C24	23.5 (8)
N3—Gd1—N7—C24	109.3 (5)	C28—N7—C24—C25	−0.3 (10)
N9—Gd1—N7—C24	−161.9 (5)	Gd1—N7—C24—C25	178.8 (6)
C30—N8—N9—C32	2.7 (7)	C28—N7—C24—C23	176.9 (6)
C28—N8—N9—C32	−168.8 (5)	Gd1—N7—C24—C23	−4.0 (7)
C30—N8—N9—Gd1	−141.2 (4)	O6—C23—C24—N7	171.6 (6)
C28—N8—N9—Gd1	47.2 (6)	O5—C23—C24—N7	−11.0 (9)
O3—Gd1—N9—C32	−31.7 (8)	O6—C23—C24—C25	−11.2 (11)
O1—Gd1—N9—C32	39.9 (7)	O5—C23—C24—C25	166.2 (7)
O5—Gd1—N9—C32	−169.1 (6)	N7—C24—C25—C26	−0.1 (12)
N4—Gd1—N9—C32	93.8 (7)	C23—C24—C25—C26	−177.1 (7)
N7—Gd1—N9—C32	−160.7 (7)	C24—C25—C26—C27	−0.3 (12)
N1—Gd1—N9—C32	−24.9 (7)	C25—C26—C27—C28	1.0 (12)
N6—Gd1—N9—C32	120.2 (7)	C24—N7—C28—C27	1.2 (10)
N3—Gd1—N9—C32	−86.9 (7)	Gd1—N7—C28—C27	−177.9 (5)
O3—Gd1—N9—N8	97.7 (4)	C24—N7—C28—N8	179.4 (6)
O1—Gd1—N9—N8	169.4 (4)	Gd1—N7—C28—N8	0.3 (8)
O5—Gd1—N9—N8	−39.6 (4)	C26—C27—C28—N7	−1.5 (11)
N4—Gd1—N9—N8	−136.7 (3)	C26—C27—C28—N8	−179.6 (7)
N7—Gd1—N9—N8	−31.2 (3)	C30—N8—C28—N7	155.9 (7)
N1—Gd1—N9—N8	104.6 (4)	N9—N8—C28—N7	−34.9 (8)
N6—Gd1—N9—N8	−110.3 (4)	C30—N8—C28—C27	−25.9 (11)
N3—Gd1—N9—N8	42.6 (4)	N9—N8—C28—C27	143.3 (6)
O3—Gd1—O1—C1	58.6 (5)	N9—N8—C30—C31	−1.7 (8)
O5—Gd1—O1—C1	135.4 (5)	C28—N8—C30—C31	167.9 (7)
N4—Gd1—O1—C1	125.0 (6)	N9—N8—C30—C29	176.0 (6)
N7—Gd1—O1—C1	−112.9 (5)	C28—N8—C30—C29	−14.4 (12)
N1—Gd1—O1—C1	−16.3 (5)	N8—C30—C31—C32	−0.1 (8)
N6—Gd1—O1—C1	−172.1 (6)	C29—C30—C31—C32	−177.6 (7)
N3—Gd1—O1—C1	−11.4 (6)	N8—N9—C32—C31	−2.7 (7)
N9—Gd1—O1—C1	−84.7 (5)	Gd1—N9—C32—C31	128.0 (6)
O1—Gd1—O3—C12	62.6 (5)	N8—N9—C32—C33	173.5 (6)
O5—Gd1—O3—C12	−79.8 (5)	Gd1—N9—C32—C33	−55.8 (10)
N4—Gd1—O3—C12	−13.5 (4)	C30—C31—C32—N9	1.8 (9)
N7—Gd1—O3—C12	−125.5 (5)	C30—C31—C32—C33	−174.0 (7)

### Hydrogen-bond geometry (Å, °)

**Table:**

*D*—H···*A*	*D*—H	H···*A*	*D*···*A*	*D*—H···*A*
O7—H7D···O8	0.85	2.23	3.074 (19)	172
O7—H7E···O2^i^	0.85	2.07	2.914 (13)	172
O8—H8A···O4^ii^	0.85	1.96	2.740 (11)	153
O8—H8B···O11	0.85	2.15	2.93 (2)	153
O9—H9A···O8	0.85	1.81	2.633 (18)	162
O9—H9B···O6^iii^	0.85	1.94	2.765 (17)	162
O10—H10A···O7	0.85	2.07	2.64 (2)	124
O11—H11···O2^i^	0.82	2.14	2.664 (17)	122
O12—H12A···O6^iv^	0.85	2.15	2.89 (3)	146
O12—H12B···O9^v^	0.84	1.74	2.49 (5)	147
O12—H12B···O8^v^	0.85	2.47	3.12 (4)	133

Symmetry codes: (i) *x*−1/2, −*y*+1/2, *z*−1/2; (ii) −*x*+1, −*y*, −*z*+2; (iii) −*x*+1/2, *y*+1/2, −*z*+3/2; (iv) −*x*+1, −*y*, −*z*+1; (v) *x*+1/2, −*y*+1/2, *z*−1/2.
